# Aggressiveness, ADHD-like behaviour, and environment influence repetitive behaviour in dogs

**DOI:** 10.1038/s41598-022-07443-6

**Published:** 2022-03-24

**Authors:** Sini Sulkama, Milla Salonen, Salla Mikkola, Emma Hakanen, Jenni Puurunen, César Araujo, Hannes Lohi

**Affiliations:** 1 Department of Veterinary Biosciences, University of Helsinki, PL 63 (Haartmaninkatu 8), 00014 Helsinki, Finland; 2 Department of Medical and Clinical Genetics, University of Helsinki, Helsinki, Finland; 3 Folkhälsan Research Center, Helsinki, Finland

**Keywords:** Behavioural ecology, Ecology, Psychiatric disorders, Obsessive compulsive disorder

## Abstract

Repetitive behaviour ranges from variants of normal repetitive behaviours to abnormal repetitive behaviours. The domestic dog spontaneously performs different repetitive behaviours, which can be severe and impair the quality of life and the dog-owner relationship. We collected comprehensive behavioural questionnaire data from almost 4500 Finnish pet dogs and studied the effect of several demographic, environmental, and behavioural factors on canine repetitive behaviour with logistic regression. We replicated findings from previous studies by revealing comorbidity between repetitive behaviour and behavioural factors aggressiveness, hyperactivity/impulsivity, and inattention. We also found a novel association between repetitive behaviour and the owner’s dog experience. In addition, we showed that repetitive behaviour is more common in dogs that live without conspecifics, dogs that were given a low amount of exercise, dogs that lived in larger families, young dogs and elderly dogs, and neutered dogs. Finally, we identified breed differences in repetitive behaviour, suggesting that some breeds are more vulnerable to repetitive behaviour and indicate a genetic susceptibility. As abnormal repetitive behaviour can considerably worsen the well-being of dogs and impair the dog-owner relationship, a better understanding of the environmental, lifestyle, and molecular factors affecting canine repetitive behaviour can benefit both dogs and humans.

## Introduction

Repetitive behaviour includes invariant, repetitive behaviour patterns ranging from variants of normal repetitive behaviours to abnormal repetitive behaviours. Abnormal repetitive behaviours are seen in wild and domesticated captive animals but not in nature, although they seem to be the product of normal behavioural processes^1^. Examples of repetitive behaviour patterns in animals include feather picking in parrots^2^, pacing in zoo-housed polar bears^3,4^, tigers^5^ and lions^3^, and crib-biting in horses^6^. Repetitive behaviours are also common in pets^7,8^. Terminology concerning inappropriate, invariant, repetitive behaviour is not well established: several terms such as abnormal repetitive behaviour, stereotypic behaviour, compulsion and compulsive behaviour have been used. To clarify the differences, abnormal repetitive behaviours can be divided into stereotypies and compulsions based on what is repeated^9,10^. Stereotypies are repetitions of certain motor patterns with no apparent goal or function, whereas compulsions are repetitions of a certain inappropriate goal^11,12^. Despite the differences, they are often difficult to distinguish^10^. Here, we chose to use the term repetitive behaviour, as we do not know if the behaviour is abnormally repeated and whether the behaviour has a function or a goal.

In the domestic dog, spontaneous repetitive behaviours occur in many forms. Repetitive behaviour patterns are observed in 16% of pet dogs^8^. Canine abnormal repetitive behaviours have been categorised as locomotory (circling, tail chasing, pacing, chasing light reflections, freezing), oral (leg or foot chewing, self-licking, flank sucking, chewing or licking of objects, and snapping in the air (fly snapping)), aggressive (self-directed aggression, growling or biting the rear end, rear legs, or tail), vocalization (compulsive rhythmic barking or whining), and hallucinatory behaviours (staring at shadows and chasing light reflections)^11^. Repetitive behaviour typically starts during puppyhood before the age of one year^13,14^. It varies in severity and duration, and is often triggered by frustration, boredom or stress^14–16^. As the performance of repetitive behaviours increases, they can also become generalised to many contexts^17^, and become more challenging to interrupt, making the repetitive behaviour more persistent^10^. Ultimately, severe repetitive behaviour may considerably worsen the quality of life^14^ and the dog-owner relationship^16^.

The origin of repetitive behaviour is complex, contributed by both environmental and genetic factors. Early weaning age^7^, lack of socialisation, and presence of conspecifics^14^ have been linked to repetitive behaviours. Health issues and pain can be underlying causes as well^2,14,18^. Furthermore, comorbidity between repetitive behaviours and other behavioural problems have been reported in dogs and other animals^7,13,14,16,18^. Additionally, heritable contributions have been demonstrated by the observed breed-specificity of repetitive behaviours, as well as recent gene discoveries^14,19–22^.

Obsessive–compulsive disorder (OCD) in humans is a severe psychiatric disorder^23,24^. Neuroimaging and genetic studies have linked OCD with the cortico–striato–thalamo–cortical (CSTC) system loops^25^ mediated by serotonergic, glutaminergic and dopaminergic neurotransmitter systems^26^. Canine compulsive disorder, which is characterized by abnormal repetitive behaviours has been proposed as a model for human OCD^27,28^, as a growing body of evidence shows many similarities between human and canine compulsions. These include early age of onset^13,14,29,30^, behavioural inflexibility resulting from executive function impairment^31–33^, structural abnormalities in the brain^34,35^, increased blood cholesterol levels^36^, imbalanced serotonergic and dopaminergic pathways^26,37^, and similar pharmacological and non-pharmacological interventions for treatment^10,36,38,39^. In dogs, fluoxetine^39^ and clomipramine^13,36,38^ are used to treat compulsive disorders with repetitive behaviours, and both are commonly used to treat human OCD as well. However, the equivalence of canine and human compulsions has been challenged^40^. Different forms of OCD and canine compulsive disorder probably involve some different factors, but it can be suggested that different compulsions also overlap and share biological etiology and common genetic factors^13,14,16^.

This study utilised a comprehensive questionnaire-based approach to explore the demographic, environmental, and behavioural factors associated with canine repetitive behaviour in almost 4500 Finnish pet dogs. Identifying associated risk factors of repetitive behaviour could help prevent the welfare and management problems related to these traits and develop a strategy for robust genetic studies with more susceptible breeds.

## Results

### Study cohort and demographics

We studied the effects of environmental, demographic, and behavioural factors on canine repetitive behaviour with an owner-completed online questionnaire. We collected a study cohort of 4436 dogs, including 1315 dogs displaying repetitive behaviours and 3121 dogs not showing any repetitive behaviour. 54% of the dogs were female. The age of the dogs varied from 2.4 months to 17.9 years, with a mean of 4.8 years (SD ± 3.3). More detailed demographics are presented in Supplementary Table S2.

### Factors associated with repetitive behaviour

The best model explaining the differences in the probability of repetitive behaviour included several demographic, environmental, and behavioural variables, such as age, sex, breed, sterilisation status, owner’s dog experience, number of dogs in the family, family size, daily exercise, urban environment score, hyperactivity/impulsivity score, inattention score, and aggressiveness.

The age of the dog was associated with repetitive behaviour (Table 1, Supplementary Fig. S1a). The probability of repetitive behaviour was higher in young dogs, decreased with age until 8 years of age, and then again increased in elderly dogs (linear effect: F = 9.55, df = 1, p = 0.0183; quadratic effect: F = 6.68, df = 1, p = 0.0543). Contrary to our a priori hypothesis, there was no significant difference in repetitive behaviour between male and female dogs (OR 0.954, df = 1, p = 0.5322). Instead, there was an association between sterilisation and repetitive behaviour, as intact dogs had a lower probability of repetitive behaviour than neutered dogs (OR 0.706, df = 1, p = 0.0020).

**Table 1:** Associations of the demographic, environmental, and behavioural variables with repetitive behaviour in the logistic regression analysis.

Variable	F	DF	p value
Age	9.55	1	**0.0183**
Age^2	6.68	1	0.0543
Sex	0.39	1	0.5336*
Breed	3.29	23	** < 0.0001**
Sterilisation	15.33	1	**0.0020**
Dogs in the family	34.64	1	< 0.0001*
Daily exercise	7.79	3	**0.0018**
Owner’s dog experience	26.85	1	** < 0.0001**
Family size	4.20	4	0.0183
Urban environment score	3.75	1	0.1622
Urban environment score^2	2.23	1	0.2797
Aggressiveness	24.36	2	** < 0.0001**
Hyperactivity/impulsivity	147.71	1	** < 0.0001**
Inattention	31.02	1	** < 0.0001**

P-values, except a priori contrasts, are controlled for false discovery rate. Variables for which a priori contrasts were set and which p-values are not false discovery controlled are denoted with *. Significant effects are indicated in bold (p-value < 0.05). N = 4436.

**Figure 1:**
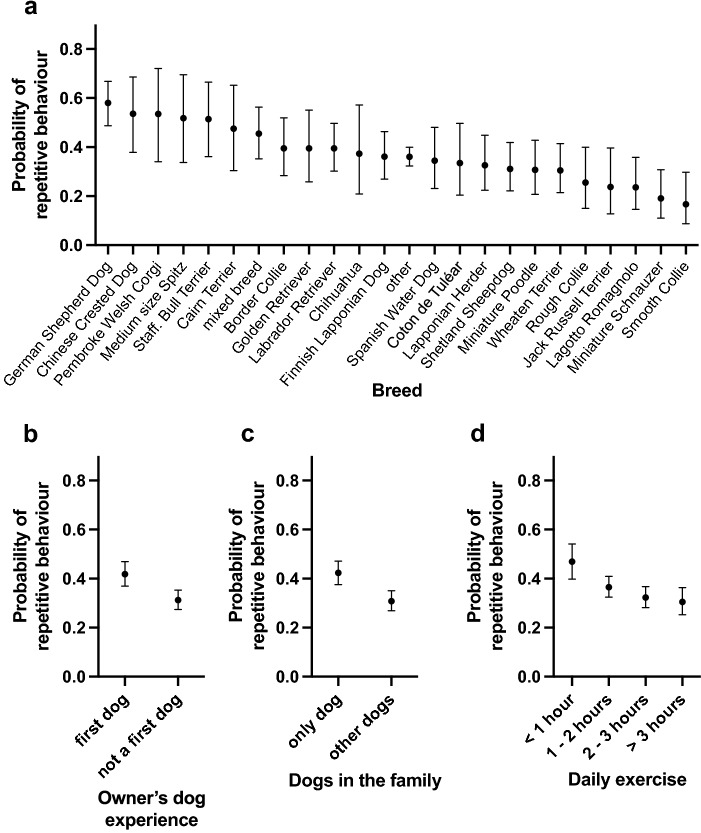
The effects of breed (**a**), owner’s dog experience (**b**), number of dogs in the family (**c**), and daily exercise (**d**) on the probability of repetitive behaviour in the logistic regression analysis. Error bars indicate 95% confidence limits. N = 4436.

Several environmental factors were associated with repetitive behaviour. Dogs getting less daily exercise had a higher probability of repetitive behaviour (Table 1, Fig. 1d). More specifically, dogs getting less than one hour of exercise per day had a higher probability of repetitive behaviour than dogs exercising 1–2 h (OR 1.53, df = 1, p = 0.0183), 2–3 h (OR 1.85, df = 1, p = 0.0020), or more than three hours (OR 2.01, df = 1, p = 0.0020) per day. As hypothesised, dogs that were the only dogs in the family had a higher probability of repetitive behaviour than dogs living with other dogs (OR 1.64, df = 1, p < 0.0001) (Table 1, Fig. 1c). The owner’s dog experience was also associated with the probability of repetitive behaviour (Table 1, Fig. 1b). If the dog was the owner’s first dog, it was more likely to have repetitive behaviour than if it was not the owner’s first dog (OR 1.58, df = 1, p < 0.0001). In addition, family size was associated with repetitive behaviour (Table 1, Supplementary Fig. S1e). Dogs living in single-person households (“single”) were less likely to show repetitive behaviour than dogs living in two-person households (“couple”) (OR 0.687, df = 1, p = 0.0034) or in larger families (more than two children or more than two adults in the family) (OR 0.672, df = 1, p = 0.0225). Urban environment score was not associated with the probability of repetitive behaviour (linear effect: F = 3.75 , df = 1, p = 0.1622, quadratic effect: F = 2.23 , df = 1, p = 0.2797) (Table 1, Supplementary Fig. S1b).

We examined breed differences in repetitive behaviour in 22 breeds. We also included a group consisting of all the other breeds in the data (“other”) and mixed breed dogs. The logistic regression analysis detected differences between breeds in repetitive behaviour (Table 1), with the highest probability of repetitive behaviour observed in German Shepherd Dogs, Chinese Crested Dogs, Pembroke Welsh Corgis, Medium size Spitzes, and Staffordshire Bull Terriers. The breeds with the lowest probability of repetitive behaviour were Smooth Collie, Miniature Schnauzer, Lagotto Romagnolo, Jack Russel Terrier, and Rough Collie (Fig. 1a). The largest pairwise differences were found between Smooth Collie and German Shepherd Dog (OR 0.145, df = 1, p = 0.0020), Miniature Schnauzer and German Shepherd Dog (OR 0.170, df = 1, p = 0.0020), Smooth Collie and Chinese Crested Dog (OR 0.174, df = 1, p = 0.0056), and Smooth Collie and Pembroke Welsh Corgi (OR 0.174, df = 1, p = 0.0143). All pairwise breed differences are presented in Supplementary Dataset and significant pairwise breed differences in Supplementary Table S5. As we hypothesised a priori, German Shepherd Dog and Staffordshire Bull Terrier had a significantly higher probability of repetitive behaviour when compared with other breeds (OR 2.27, df = 1, p < 0.0001).

**Figure 2:**
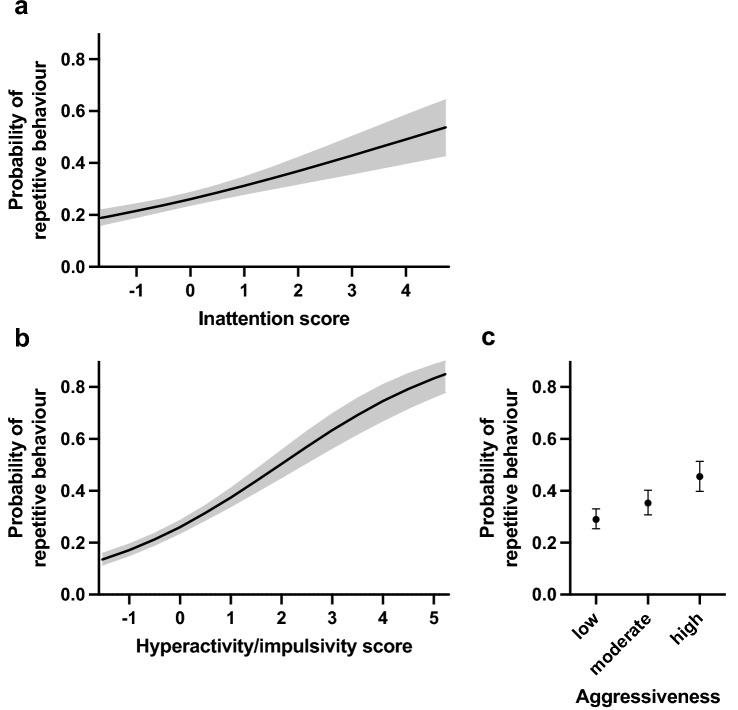
The effects of inattention score (**a**), hyperactivity/impulsivity score (**b**), and aggressiveness (**c**) on the probability of repetitive behaviour in the logistic regression analysis. Grey area (**a, b**) and error bars (**c**) indicate 95% confidence limits. N = 4436.

In addition, behavioural factors were positively associated with repetitive behaviour. Dogs with higher hyperactivity/impulsivity scores (F = 147.71, df = 1, p < 0.0001) and higher inattention scores (F = 31.02, df = 1, p < 0.0001) had a higher probability of repetitive behaviour (Table 1, Fig. 2a,b). Furthermore, aggressiveness increased the probability of repetitive behaviour (Table 1, Fig. 2c). As we hypothesised, dogs with high levels of aggressiveness had a higher probability of repetitive behaviour than dogs with low aggressiveness (OR 2.04, df = 1, p < 0.0001). Moreover, dogs reported to have high levels of aggressiveness had a higher probability of repetitive behaviour than dogs with moderate levels of aggressive behaviour (OR 1.53, df = 1, p = 0.0034), and dogs with moderate levels of aggressive behaviour had a higher probability of repetitive behaviour than the dogs with no reported aggressiveness (OR 1.33, df = 1, p = 0.0116).

## Discussion

We have performed an extensive survey-based study on canine repetitive behaviour with almost 4500 dogs, identifying associated demographic, environmental, and behavioural factors. Using a dataset combining different forms of repetitive behaviour, we demonstrate behavioural comorbidities, suggest new associations, and indicate a considerable overlap with the previously reported risk factors of canine repetitive behaviours.

The probability of repetitive behaviour was significantly higher in dogs reported by their owners to have high aggressiveness, which is in line with most previous canine studies. For example, Overall et al.^13^ reported that 75% of compulsive dogs had concurrent behaviour problems and 28% of compulsive dogs had dominance/impulse control aggression. Moon-Fanelli et al.^16^ reported increased aggression in tail-chasing Bull Terriers, although the study by Tiira et al.^14^ found the opposite. The connection between repetitive behaviour and aggressiveness has also been reported in cats^7^ and rhesus macaques^18^. Thus, repetitive behaviour may be correlated with particular behavioural or personality traits.

The probability of repetitive behaviour increased with increasing scores in hyperactivity/impulsivity and inattention. Repetitive behaviour was previously associated with impulsivity^41^ and hyperactivity^42^, and the same connection was also found in our two previous papers^8,43^. We also observed comorbidity between inattention and repetitive behaviour. Inattention has been little studied in dogs, and this observed comorbidity has previously been described only in our two previous articles of this same but expanded dataset^8,43^. In this study, these comorbidities observed in our previous articles persisted despite the inclusion of different demographic and environmental variables. Based on the observed comorbidities, it can be speculated whether repetitive behaviour patterns are simply bursts of energy or signs of abnormal repetive behaviour.

Our results agree with current literature on human OCD and behavioural comorbidities. For example, high comorbidity between OCD and ADHD has been detected in humans, and both conditions are characterised by impaired inhibitory control and deficits in executive function^26,35,44–46^. Impulsive aggression has long been linked to OCD, and the occurrence of aggression is common in both compulsive and impulsive disorders^47^. These comorbidities likely result from partially shared neurobiological loops and brain structures involved in these conditions, and they may also share some common genetic factors.

We identified several environmental factors associated with repetive behaviours. As a novel finding, we discovered that repetitive behaviour was more common in dogs that were their owners’ first dogs. Repetitive behaviour is increased by stress, and thus, a predictable environment may decrease canine compulsive disorders^10^. Inexperienced owners may provide inconsistent training, which could increase stress, possibly explaining our results. Another possible hypothesis is that inexperienced owners may not detect abnormal repetitive behaviour as early as experienced owners, and with repetition, canine repetitive behaviours are more likely to persist^10^. However, a replication study would be warranted to solidify this finding.

Dogs that were the only dog in the family had a higher probability of repetitive behaviour than dogs living with other dogs. Similarly, Tiira et al.^14^ found that dogs living with many other dogs chased their tails less than dogs living alone or with fewer dogs, but this was only observed in Bull Terriers. Also, in sheep (*Ovis aries*), stereotypies were more common in single-housed than group-housed sheep^48^. Tiira et al.^14^ suggested that conspecifics in the same household may reduce frustration and boredom, potential risk factors for repetitive behaviours^16^.

Low daily exercise increased the probability of repetitive behaviour in dogs. This result contradicts an earlier study that found no significant association between exercise and tail chasing^14^. In contrast, a previous study based on YouTube video material suggested that tail chasing might result from a lack of activities, exercise or stimulation^49^. However, in humans, physical activity may prevent anxiety disorders, but it can also be used as a treatment to improve stress resilience and decrease anxiety^50^. It has been proposed that frustration and stress contribute to canine compulsive disorders^11^, and exercise has been recommended as a behaviour modification technique^11,51^.

In our study, the probability of repetitive behaviour was higher in dogs living in two-person households or larger families when compared with single-person households. In contrast, Tiira et al.^14^ found that in Staffordshire Bull Terriers, dogs living in households with more children chased their tails less than dogs living with fewer or no children. They also found no association between the number of adults in the household and tail chasing. The aforementioned stress-related association with canine repetitive behaviours^10^ could also explain our finding of an association with family size. In larger families, potentially with a noisier and busier environment, life can be more stressful and predispose individuals to perform repetitive behaviour. It is also possible that in single-person households, owners have more time to spend with their dogs and give them attention, such playtime, petting, and exercise that can also reduce stress. Because no previous results report a similar association with our results, the possible relationship between these factors needs to be further validated.

Multiple demographic factors were associated with repetitive behaviour in dogs, including age and sterilisation. We found that repetitive behaviour was most common in young dogs and elderly dogs. Paralleling our results, Tiira et al.^14^ observed that tail chasing typically starts at the age of 3–6 months in Bull Terriers, and in another study, the mean age of onset of tail chasing was 6 months^16^. Temporary tail chasing is typically seen in puppies, but sometimes it can continue after puppyhood. Occasionally, senior dogs (aged > 8 years) also show behaviours similar to repetitive behaviours. Canine cognitive dysfunction, which exhibits symptoms resembling dementia or Alzheimer’s disease in humans, may cause repetitive behaviours (such as licking, inattentiveness or staring), but this is a consequence of a progressive neurological disorder^52^. This may explain the increase in repetitive behaviour in older dogs. Our canine data results resembled the results of Delorme et al.^53^, where they discovered human OCD to have a bimodal age distribution, with the first peak in adolescence and a second in early adulthood.

Previous research suggests that obsessive–compulsive disorder is more common in male dogs compared to females^13^. Similarly, Moon-Fanelli et al.^16^ found that males have a slightly higher probability of tail chasing than females in Bull Terriers. In contrast, we did not find a significant difference between the sexes in repetitive behaviour. Instead, we observed a higher probability of repetitive behaviour in neutered dogs than in intact dogs. Contrary to our study, Tiira et al.^14^ found that sterilized individuals, especially females, have less severe tail chasing compared to intact dogs and hypothesised that in females this could be an effect of reduction in the production of progesterone and oestradiol, which may have a controlling effect on compulsions. However, in many animal studies, low levels of ovarian hormones have worsened the symptoms of repetitive behaviours^54,55^. In the case of contradictory findings between different studies, the causality between sterilisation and repetitive behaviour can only be speculated. It is not clear whether repetitive behaviour is more common in neutered dogs or whether the dogs are neutered because of unwanted behaviour.

We observed differences in the prevalence and type of compulsions between breeds, suggesting a genetic contribution. The breeds with the highest probabilities of repetitive behaviour include German Shepherd Dog, Chinese Crested Dog, Pembroke Welsh Corgi, Medium size Spitz, and Staffordshire Bull Terrier. Repetitive behaviour was most often reported in German Shepherd Dogs, and similarly, Col et al.^56^ reported that German Shepherd Dogs were at increased risk of obsessive behaviour. However, besides showing differences in the probability of any repetitive behaviour, breeds also display different compulsions. In this present study, all subtraits of repetitive behaviour were combined, but in our previous exploration^8^ of this same but expanded dataset, we observed that, for example, Staffordshire Bull Terriers had a high prevalence of tail chasing. In contrast, Border Collies displayed a very high prevalence of compulsive staring and fly snapping. Other previous studies have also identified certain breeds that are more susceptible to specific repetitive behaviours than others^11,20,49,57,58^. It is possible that as different dog breeds have their breed-typical characteristics and functional purposes, certain behaviours become more common and dominant in certain breeds. However, it is important to note that in different studies, the results are based on the breeds available for a specific study cohort. In many studies, only a particular repetitive behaviour is studied. Thus, comparing the findings of different studies may be difficult.

This study has limitations. First, different forms of repetitive behaviour may have a different neurobiological basis^10^, but we could not separate these because of the small sample sizes in the studied subtraits of repetitive behaviour. This should be noticed when considering our results. However, in their study, Cao et al.^59^ suggested that the gene area earlier linked to flank sucking (CDH2) in Doberman Pinchers^60^ would also be associated with tail chasing in Belgian Shepherd Malinois. In many previous studies, only one specific repetitive behaviour has been studied, making comparisons between these studies and our study challenging. Second, repetitive behaviour can be caused by other factors than actual compulsion. We did not collect any health information, and thus we could not identify individuals with possible health problems. Third, certain risk factors of repetitive behaviour can be linked to each other and sometimes repetitive behaviours may be difficult to distinguish from other behaviours, confounding our results. Fourth, our study is based on a questionnaire, and participation was voluntary. Questionnaires can be an effective way of collecting data, but they can also be subjective. Our data is a self-selected convenience sample and may not be a representative sample of the overall Finnish pet dog population. However, questionnaires have been indicated to be helpful in behavioural science as their reliability and validity are good and questionnaire answers strongly associate with the behaviour of the animals^61^. In the future, it would be important to separate the different forms of repetitive behaviour, collect larger sample sizes of each subtrait, and collect comprehensive health information from dogs.

In conclusion, we showed that canine repetitive behaviour is a complex entity associated with several demographic, environmental, and behavioural factors. We replicated findings from previous studies by identifying comorbidity between repetitive behaviour and other behavioural factors: aggressiveness, hyperactivity/impulsivity, and inattention. Interestingly, we reported a novel association between repetitive behaviour and the owner’s dog experience. Moreover, we observed that a low amount of exercise and larger family size, environmental factors potentially increasing stress in the dogs’ life, may increase the probability of repetitive behaviour. Our results also replicated findings from previous research on canine repetitive behaviour, as we showed that repetitive behaviours are more common in dogs that live without conspecifics. We also identified that repetitive behaviour is typically seen in young dogs and elderly dogs, and in neutered dogs. In addition, we observed differences between dog breeds in repetitive behaviour, suggesting that some breeds may be more vulnerable to develop problems related to repetitive behaviour and indicating genetic susceptibility. As abnormal repetitive behaviour can considerably worsen the well-being of dogs and impair the dog-owner relationship, understanding the factors affecting canine repetitive behaviours can benefit both dogs and humans.

## Material and methods

### Data collection

#### Questionnaire

We designed an online owner-completed behavioural questionnaire to collect extensive behavioural data and background information from a considerable number of Finnish pet dogs. The questionnaire consisted of questions about seven different canine behavioural traits: fear, aggressiveness, noise sensitivity, fear of surfaces and heights, hyperactivity/impulsivity and inattention, separation-related behaviour, and repetitive behaviour. It also included a large background section covering demographic and environmental questions related to each dog’s life history. The questionnaire was advertised for all breeds on Facebook, on the research group’s web pages and via breed clubs. Questionnaire replies were collected from February 2015 to September 2018. The questionnaire and more details about behavioural trait categorisation can be found as Supplementary material in Salonen et al.^8^. Here, we studied the demographic, environmental, and behavioural factors associated with repetitive behaviour in dogs.

#### Repetitive behaviour

To assess repetitive behaviour of dogs, we asked dog owners to estimate the occurrence of several subtraits of repetitive behaviour. Tail chasing, reflection/shadow snatching, surface licking, pacing, and staring were estimated on a Likert-type scale from 0 (I have never noticed this behaviour) to 6 (I have noticed this behaviour several times per day). The dogs scoring from 4 (every other day-weekly) to 6 were categorised into the high group, and dogs scoring 0 or 1 (a few times during the dog’s lifetime) were categorised into the low group. We also asked owners to estimate the time dog spends near the water bowl from less than 5 min to 1 h or more (indicating water bowl compulsion). Dogs that spent less than 5 min near the water bowl formed the low group, and dogs that spent more than 15 min near it formed the high group. In addition, the incidence of self-biting was rated on a scale from 0 (never) to 3 (several hours per day), and dogs scoring 2 (almost every day) or 3 composed the high group, whereas dogs scoring 0 composed the low group. Finally, we considered data from all the subtraits together. A dog was classified into the low group in repetitive behaviour if it was categorised into the low group in all subtraits of repetitive behaviour and into the high group if categorised into the high group in at least one subtrait. The dogs not meeting these criteria were categorised into the moderate group. As we used logistic regression in the analysis, dogs categorised into the moderate group were excluded. Detailed information about the questions can be found in the Supplementary information.

#### Demographic, behavioural, and environmental variables

Before statistical analyses, we edited some demographic and environmental variables derived from the behavioural questionnaire. We created some new variables already described in our previous articles^43,62–64^.

We selected 22 dog breeds with adequate sample sizes (< 10 individuals/group) in both high and low groups. Individuals in other breeds with inadequate sample sizes were combined under the breed group “other”. Mixed breed dogs were also included in the data. To quantify the environmental land-use around the dog’s home, we created a continuous variable “urban environment score” using addresses provided by the dog owners. Geographical coordinates for each home were derived using address information. The proportion of three different land-use types (artificial surfaces, agricultural areas, and forests and semi-natural areas) within a three-kilometre range was defined using a public land-use database CORINE2012 with a 25-m resolution. This land-use information was further simplified into one continuous rural–urban gradient using principal component analysis. Higher values of built environment correlated with a higher urban environment score.

We also utilised a variable “family size” with five categories: one adult living alone (“single”), childless couple (“couple”), one-child family with one to two adults (“one child”), two-children family with one to two adults (“two children”), a bigger family with more than two children or more than two adults (“larger family”). We included a variable “dogs in the family” describing the presence of conspecifics: either the dog was an only dog, or the owner had other dogs as well. We also included a variable, “owner’s dog experience” describing whether the dog was the owner’s first dog or not. Moreover, we had a variable “daily exercise” to illustrate the amount of exercise (not including spending time alone in the yard) that was categorised into four categories: less than 1 h per day, 1 to 2 h per day, 2 to 3 h per day, and over 3 h per day.

Additionally, we created continuous behavioural variables “hyperactivity/impulsivity” and “inattention” and a categorical behavioural variable “aggressiveness”. To measure individual differences in hyperactivity/impulsivity and inattention, we used the dog ADHD survey developed and validated by Vas et al.^65^. The survey included 13 statements (described in our previous article by Sulkama et al.^43^), and the dog owners were asked to answer how often the statement is true for their dog. A principal component analysis divided the questionnaire statements into two components, hyperactivity/impulsivity and inattention. We calculated the component scores of hyperactivity/impulsivity and inattention for each dog, with higher component scores indicating a higher level of hyperactivity/impulsivity or inattention. Aggressiveness included two subtraits; owners were asked to rate the likelihood of their dog displaying aggressive behaviour towards strangers and family members. The signs of aggressive behaviour were snapping or biting and growling. In aggressiveness, dogs were divided into three groups: low, moderate, and high. The low group included dogs that never showed aggressive behaviour. The moderate group included dogs that showed aggressiveness no more than occasionally. The high group included the dogs with regular aggressive behaviour in either one of the subtraits.

All explanatory variables derived from the behavioural questionnaire are explained in detail in Supplementary Table S1.

### Statistical analyses

All statistical analyses were conducted in R version 3.6.2^66^. We used a logistic regression model to study the association between canine repetitive behaviour and the demographic, environmental and behavioural variables chosen based on previous literature. We combined subtraits of repetitive behaviour for the analysis due to a small number of dogs displaying repetitive behaviour in many breeds. Initially, we selected seventeen explanatory variables mostly based on previous literature: age, sex, breed, sterilisation, body size (as demographic explanatory variables), daily exercise, number of dogs in the family, urban environment score, family size, owner’s dog experience, socialisation score, daily time spent alone, weaning age (as environmental explanatory variables), hyperactivity/impulsivity, inattention, fearfulness, and aggressiveness (as behavioural explanatory variables) (Supplementary Table S1). The initial questionnaire data consisted of 13,715 dogs in 264 breeds. After excluding individual dogs with missing or incomplete responses in the studied explanatory variables, the data included 3460 dogs.

We used a forward stepwise AIC (Akaike Information Criterion) model selection approach, adding variables one by one to find the models with the best fit. The model selection was initiated with a model that included age and sex as explanatory variables, as dogs of different sexes and ages differed in prevalence in our previous study^8^. The model selection process favoured the inclusion of the explanatory variables breed, sterilisation, owner’s dog experience, number of dogs in the family, family size, daily exercise, urban environment score, hyperactivity/impulsivity score, inattention score, and aggressiveness which improved the model fit, and thus, they were included in the final model. In contrast, the explanatory variables body size, fearfulness, socialisation score, weaning age, and time spent alone did not improve model fit and were excluded. The AIC model selection and the final model are presented in Supplementary Table S4. To maximise the sample size, we created a new subset of the initial data after model selection by including all dogs with missing responses only in the explanatory variables that were not selected in the final model. As a result, the final dataset consisted of 4436 individuals.

After the model selection, we checked the linearity assumptions of continuous explanatory variables by fitting a generalized additive model with the package “gam”^67^ in R. We included both linear and quadratic variables (e.g. age and age^2^) in the final model if the assumption was not met. Next, we examined possible outliers with packages “broom”^68^ and “dplyr”^69^. We plotted standardised residuals using the package “ggplot2”^70^. Then we tested multicollinearity by requesting the generalised variance inflation factor (gVIF) with the package “car”^71^ in R. There was no multicollinearity and outliers were not found. Finally, we calculated the area under the receiver operator characteristic curve (AUC) using package “pROC”^72^. The AUC of the final model was 0.77, indicating good discrimination ability.

To obtain the overall effects of the explanatory variables on repetitive behaviour, we conducted an analysis of variance (ANOVA) with the package “car”^71^ in R. Furthermore, we calculated the estimated marginal means for categorical explanatory variables with the package “emmeans”^73^ in R. We obtained the effects of continuous explanatory variables (adjusting for other variables in the models) with the package “effects”^74^.

Based on previous studies, we had many hypotheses, and we formed several a priori contrasts between the levels of explanatory variables. We hypothesised that males would have a higher probability of repetitive behaviour than females^16^. We also hypothesised that Staffordshire Bull Terrier and German Shepherd Dog would have higher probabilities of repetitive behaviour than other breeds^14,56,75^. Additionally, we hypothesised that only dogs would differ from dogs living with other dogs^14^. Furthermore, we hypothesised that dogs showing high aggressiveness would have higher probabilities of repetitive behaviour than dogs not showing aggressiveness^16^.

We examined a priori contrasts, and all pairwise comparisons between levels of the included categorical variables with the package “emmeans”^73^. As we had many categorical variables, the number of pairwise comparisons was high, and therefore, all p-values of the logistic regression analysis, except contrasts chosen a priori, were controlled for false discovery rate (FRD) to decrease the probability of type I error. The significance cut-off was set at a p-value < 0.05. All methods were carried out in accordance with local guidelines and regulations.

### Ethics statement

The data was collected before the onset of the GDPR according to the Finnish legislation: https://www.finlex.fi/fi/laki/ajantasa/1999/19990523. This survey study focused on dogs and not their owners (human participants), and therefore an ethical approval was not needed at that time for academic research studies. We collected only the names and addresses of dog owners for the purpose of contacting the owners in subsequent studies and for calculating the urban-environment score. Informed consent was obtained from all participants. When filling the questionnaire, participants agreed that all questionnaire answers could be used for research. We stated that all data would be handled strictly confidentially and that individual dogs and owners cannot be recognised from the published results.

## Supplementary Information

Supplementary Information 1.

Supplementary Information 2.

Supplementary Information 3.

## Data Availability

The anonymised data is available as Supplementary material in the article by Salonen et al.^8^.
