# Supplementary material for: Aggressiveness, ADHD-like behaviour, and environment influence repetitive behaviour in dogs
Source: Sci Rep. 2022 Mar 24;12:3520. doi: 10.1038/s41598-022-07443-6 (PMC8948230; doi:10.1038/s41598-022-07443-6)
Supplement: Supplementary file 1 — Supplementary Information 1. [file 41598_2022_7443_MOESM1_ESM.pdf]

Aggressiveness, ADHD-like behaviour, and environment influence repetitive behaviour in dogs

Sini Sulkama, MSc[1,2,3], Milla Salonen, PhD[1,2,3], Salla Mikkola, MSc[1,2,3], Emma Hakanen, MSc[1,2,3], Jenni Puurunen, PhD[1,2,3], César Araujo, PhD[1,2,3], Hannes Lohi, PhD, prof.[1,2,3#]

[1]Department of Veterinary Biosciences, University of Helsinki, Helsinki, Finland

[2]Department of Medical and Clinical Genetics, University of Helsinki, Helsinki, Finland

[3]Folkhälsan Research Center, Helsinki, Finland

# Corresponding author:

Hannes Lohi, PhD, Professor

+358294125085

Email: hannes.lohi@helsinki.fi (HL)

PL 63 (Haartmaninkatu 8), 00014 HELSINGIN YLIOPISTO, Finland

Supplementary Table S1. Variables derived from the behavioural questionnaire data.

Variable	Explanation	Possible values
Repetitive behaviour	Binomial variable. Describes how often the dog shows repetitive behaviours. The low group included dogs that never showed repetitive behaviour, and the high group included dogs with regular repetitive behaviour.<br><br>Subtraits: tail chasing, reflection/shadow snatching, surface licking, pacing, and staring (scale from 0 = never to 6 = several times per day); water bowl compulsion (scale from less than 5 minutes to 1 hour or more); self-biting (scale from 0 = never to 3 = several hours per day).	0: low group<br>2: high group
Age	Continuous variable. The age of the dog (in years) at the time when the owner filled in the behavioural questionnaire.	0.2 – 17.9
Sex	Binomial variable. Sex of the dog.	1: male<br>2: female
Breed	Categorical variable. Breed of the dog reported by the owner. 22 breeds with adequate sample sizes were chosen. Mixed breed was also included in the data. Individuals in other breeds were combined under 'other' breed group.<br><br>For Chihuahua, both coat types were combined.<br>For Chinese Crested Dog, both coat types were combined.<br>For Miniature Poodle, Toy, Miniature, and Medium sizes were combined.	Border Collie, Cairn Terrier, Chihuahua, Chinese Crested Dog, Coton de Tuléar, Finnish Lapponian Dog, German Shepherd, Golden Retriever, Jack Russell Terrier, Labrador Retriever, Lagotto Romagnolo, Lapponian Herder, Medium size Spitz, Miniature Poodle, Miniature Schnauzer, Mixed breed, Other, Pembroke Welsh Corgi, Rough Collie, Shetland Sheepdog, Smooth Collie, Spanish Water Dog, Staff. Bull Terrier, Wheaten Terrier
Sterilisation	Binomial variable. Information whether the dog was intact or neutered.	0: intact<br>1: neutered
Body size	Categorical variable. Based on the average height of the breed. dogs were divided into three body size categories.	1: small, ≤ 35 cm<br>2: medium, 36-49 cm<br>3: large, ≥ 50 cm
Daily exercise	Categorical variable. The amount of the dog's daily exercise in hours.	1: < 1 hour<br>2: 1-2 hours<br>3: 2-3 hours<br>4: > 3 hours
Dogs in the family	Binomial variable. Describes whether there are other dogs in the family.	1: the dog is the only dog in the family<br>2: there are other dogs in the family
Urban environment score	Continuous variable. Describes the environmental land-use around the current home of the dog. The coverages of three land-use types (artificial surfaces, agricultural areas, forests, and semi-natural areas) were calculated within a 3-km range around the homes and simplified into a single continuous variable (higher values indicate a more urban environment).	-2.28 – 3.10
Family size	Categorical variable. Describes the number of people in the family.	1: single<br><br>2: couple<br><br>3: family with one child and one or two adults

		4: family with two children and one or two adults<br><br>5: family with more than two children or more than two adults
Owner’s dog experience	Binomial variable. Describes the owner’s experience with dogs.	1: the dog was the owner’s first dog<br>2: the dog was not the owner’s first dog
Socialisation score	Continuous variable. Describes the frequency of socialisation events when the dog was 7–16 weeks old. The score is a sum of the frequencies (0 = never; 1 = 1–2 times during the puppyhood; 2 = 1–2 times during the puppyhood to 2 times per month; 3 = twice a month to twice a week; 4 = twice a week to once a day; 5 = several times a day) the dog met unfamiliar men, women and children, unfamiliar adult dogs, visited city or other place with traffic and many people, and travelled by car or by bus.	0 – 35
Daily time spent alone	Categorical variable. Describes the time that the dog spent alone daily at home without the presence of people.	1: < 3 hours<br>2: 3-6 hours<br>3: 6-8 hours<br>4: > 8 hours
Weaning age	Categorical variable. The age when the dog was weaned. Dogs still living with their mothers and dogs with missing information on weaning age were excluded.	1: weaning < 7 weeks of age<br>2: weaning at 7 weeks of age<br>3: weaning at 8 weeks of age<br>4: weaning > 8 weeks of age
Aggressiveness	Categorical variable. Describes the likelihood of the dog showing aggressive behaviour.<br>Subtraits: aggression towards strangers and towards family members (scale from 1 = never to 5 = always or almost always).	0: low group<br>1: moderate group<br>2: high group
Hyperactivity/impulsivity score	Continuous (response) variable. 5 statements concerning hyperactive/impulsive behaviour. Dog owners reported how often the statement is true for their dog on a 4-point Likert scale (from 1 = never to 4 = very often). Higher component scores indicate higher levels of hyperactivity/impulsivity.	-1.54 – 5.23
Inattention score	Continuous (response) variable. 7 statements concerning inattentive behaviour. Dog owners reported how often the statement is true for their dog on a 4-point Likert scale (from 1 = never to 4 = very often). Higher component scores indicate higher levels of inattention.	-1.67 – 4.74
Fearfulness	Categorical variable. Describes how often the dog shows fear in different situations.<br>Subtraits: fear of strangers. fear of dogs. and fear of novel situations (scale from 0 = never to 5 = always).	0: low group<br>1: moderate group<br>2: high group

Supplementary Table S2. Descriptive statistics. N= 4,436

		mean	SD
Age (years)		4.79	3.26
Urban environment score		-0.03	1.40
Hyperactivity/impulsivity score		-0.02	1.06
Inattention score		-0.05	1.05
		**N**	**%**
Repetitive behaviour	Control	3,121	70.36
	Case	1,315	29.64
Sex	Female	2,376	53.56
	Male	2,060	46.44
Sterilisation	Intact	3,169	71.44
	Neutered	1,267	28.56
Breed	Border Collie	104	2.34
	Cairn Terrier	35	0.79
	Chihuahua	35	0.79
	Chinese Crested Dog	46	1.04
	Coton de Tuléar	49	1.10
	Finnish Lapponian Dog	142	3.20
	German Shepherd Dog	163	3.67
	Golden Retriever	51	1.15
	Jack Russell Terrier	48	1.08
	Labrador Retriever	155	3.49
	Lagotto Romagnolo	84	1.89
	Lapponian Herder	104	2.34
	Medium size Spitz	36	0.81
	Miniature Poodle	84	1.89
	Miniature Schnauzer	87	1.96
	Mixed Breed	116	2.61
	Other	2,535	57.15
	Pembroke Welsh Corgi	31	0.70
	Rough Collie	84	1.89
	Shetland Sheepdog	134	3.02
	Smooth Collie	69	1.56
	Spanish Water Dog	77	1.74
	Staffordshire Bull Terrier	49	1.10
	Wheaten Terrier	118	2.66
Owner's dog experience	First dog	1,077	24.28
	Not a first dog	3,359	75.72
Dogs in the family	Only dog in the family	1,461	32.94
	Other dogs in the family	2,975	67.06
Family size	Single	945	21.30
	Couple	1,831	41.28
	Family with one child and one or two adults	528	11.90
	Family with two children and one or two adults	548	12.35
	Family with more than two children or more than two adults	584	13.17
Daily exercise	< 1 hour	335	7.55

	1-2 hours	1,734	39.09
	3-4 hours	1,726	38.91
	> 3 hours	641	14.45
Aggressiveness	Low	2,665	60.08
	Moderate	1,101	24.82
	High	670	15.10

Supplementary Table S3. Contrasts between levels of categorical variables in the logistic regression analysis.

Variable	Contrasts	OR	Lower 95% Cl	Upper 95% Cl	p-value
Sex	Male vs. female	0.954	0.823	1.11	0.5322*
Owner's dog experience	The first dog vs. not the first dog	1.58	1.33	1.88	**<0.0001**
Sterilisation	Intact vs. neutered	0.706	0.594	0.840	**0.0020**
Exercise	< 1 hour vs. 1-2 hours	1.53	1.168	2.02	**0.0183**
	< 1 hour vs. 2-3 hours	1.85	1.403	2.44	**0.0020**
	< 1 hour vs. > 3 hours	2.01	1.457	2.79	**0.0020**
	1-2 hours vs. 2-3 hours	1.21	1.023	1.42	0.1031
	1-2 hours vs. > 3 hours	1.31	1.034	1.67	0.1028
	2-3 hours vs. > 3 hours	1.09	0.858	1.38	0.6457
Dogs in the family	Only dog vs. other dogs	1.64	1.39	1.93	**<0.0001***
Family size	Single vs. couple	0.687	0.563	0.839	**0.0034**
	Single vs. one child	0.849	0.648	1.112	0.4182
	Single vs. two children	0.833	0.636	1.089	0.3562
	Single vs. larger family	0.672	0.518	0.873	**0.0225**
	Couple vs. one child	1.235	0.972	1.570	0.2132
	Couple vs. two children	1.211	0.955	1.536	0.2542
	Couple vs. larger family	0.978	0.778	1.229	0.9070
	One child vs. two children	0.980	0.728	1.320	0.9391
	One child vs. larger family	0.791	0.591	1.059	0.2542
	Two children vs. larger family	0.807	0.604	1.078	0.3005
Breed	German Shepherd Dog and Staffordshire Bull Terrier vs. all other breeds	2.27	1.56	3.30	**<0.0001***
Aggressiveness	High vs. low	2.04	1.669	2.496	**<0.0001***
	High vs. moderate	1.53	1.228	1.909	**0.0034**
	Moderate vs. low	1.33	1.122	1.583	**0.0116**

P-values are controlled for false discovery rate except for *a priori* contrasts. A priori effects are denoted with*. Significant effects are emboldened (p-value < 0.05). OR = odds ratio. Cl = confidence level. N = 4,436.

Supplementary Table S4. The AIC model selection and the final models in the logistic regression analysis. N = 4,436.

**Repetitive behaviour**											
Model	AIC	Hyperactivity/ impulsivity added	Other dogs added	Aggressive-ness added	Owner's dog experience added	Inatten-tion added	Daily exercise added	Breed added	Urban environment score added	Family size added	Sterilisation added
Base model (sex + age)	4163.6										
Hyperactivity/impulsivity	**3810.6**										
Dogs in the family	4076.0	**3729.1**									
Aggressiveness	4058.0	3761.1	**3689.3**								
Owner's dog experience	4108.9	3746.0	3699.9	**3663.6**							
Inattention	3944.1	3794.3	3713.8	3676.0	**3650.0**						
Daily exercise	4132.3	3788.8	3712.4	3676.2	3651.3	**3637.9**					
Breed	4153.8	3806.8	3726.2	3685.5	3656.2	3644.1	**3632.5**				
Urban environment score	4140.1	3787.6	3722.4	3683.1	3660.7	3647.1	3635.1	**3628.9**			
Family size	4159.4	3805.2	3723.3	3685.3	3661.7	3647.5	3635.2	3628.9	**3625.2**		
Sterilisation	4140.0	3798.0	3719.6	3682.2	3658.5	3645.7	3633.8	3628.9	3626.0	**3622.4**	
Body size	4163.5	3807.8	3725.2	3687.8	3662.1	3648.4	3636.1	3632.0	3627.9	2624.4	3621.4
Fearfulness	4081.5	3795.8	3717.7	3687.4	3662.1	3649.3	3638.1	3632.2	3629.1	3625.7	3623.5
Socialisation score	4160.3	3812.0	3728.9	3690.1	3663.7	3650.8	3639.5	3634.3	3630.0	3626.5	3623.8
Weaning age	4168.5	3813.6	3732.8	3692.5	3666.4	3653.2	3641.6	3635.3	3631.8	3628.0	3625.3
Daily time spent alone	4169.5	3815.7	3734.7	3694.9	3669.4	3655.7	3643.4	3637.7	3634.1	3631.0	3628.2

Final model: sex, age, hyperactivity/impulsivity, dogs in the family, aggressiveness, owner’s dog experience, inattention, daily exercise, breed, urban environment score, family size, sterilisation.

Supplementary Table S5. Significant pairwise breed differences in the "repetitive behaviour" analysis. All p-values are FDR-controlled. All pairwise breed differences can be found in the Supplementary Dataset. OR = odds ratio. Cl = confidence limit. N = 4,436.

Breed contrast	OR	Lower 95% Cl	Upper 95% Cl	p value
Border Collie vs. Smooth Collie	3.2540	1.3582	7.7970	0.0496
Cairn Terrier vs. Smooth Collie	4.5070	1.6188	12.5470	0.0306
Cairn Terrier vs. Miniature Schnauzer	3.8410	1.4856	9.9330	0.0383
Chinese Crested Dog vs. Lagotto Romagnolo	3.7390	1.5855	8.8160	0.0215
German Shepherd Dog vs. Miniature Poodle	3.1260	1.6774	5.8270	0.0046
German Shepherd Dog vs. Finnish Lapponian Dog	2.4510	1.4349	4.1880	0.0109
German Shepherd Dog vs. Shetland Sheepdog	3.0560	1.7375	5.3750	0.0020
German Shepherd Dog vs. Wheaten Terrier	3.1530	1.7751	5.5990	0.0020
Jack Russell Terrier vs. Chinese Crested Dog	0.2690	0.1018	0.7090	0.0493
Jack Russell Terrier vs. German Shepherd Dog	0.2240	0.0990	0.5080	0.0046
Labrador Retriever vs. German Shepherd Dog	0.4730	0.2805	0.7960	0.0357
Lagotto Romagnolo vs. German Shepherd Dog	0.2230	0.1133	0.4400	0.0020
Lagotto Romagnolo vs. mixed breed	0.3690	0.1814	0.7520	0.0406
Lagotto Romagnolo vs. Pembroke Welsh Corgi	0.2680	0.1007	0.7140	0.0500
Lagotto Romagnolo vs. Staff. Bull Terrier	0.2920	0.1255	0.6780	0.0313
Lapponian Herder vs. German Shepherd Dog	0.3500	0.1907	0.6420	0.0089
Miniature Schnauzer vs. Chinese Crested Dog	0.2040	0.0842	0.4940	0.0056
Miniature Schnauzer vs. German Shepherd Dog	0.1700	0.0831	0.3490	0.0020
Miniature Schnauzer vs. German Spitz Mittel	0.2190	0.0831	0.5760	0.0183
Miniature Schnauzer vs. Labrador Retriever	0.3600	0.1726	0.7530	0.0430
Miniature Schnauzer vs. mixed breed	0.2820	0.1333	0.5950	0.0102
Miniature Schnauzer vs. other	0.4190	0.2229	0.7870	0.0440
Miniature Schnauzer vs. Pembroke Welsh Corgi	0.2040	0.0748	0.5580	0.0183
Miniature Schnauzer vs. Staff. Bull Terrier	0.2220	0.0927	0.5340	0.0098
other vs. German Shepherd Dog	0.4070	0.2823	0.5860	0.0020
Rough Collie vs. German Shepherd Dog	0.2480	0.1183	0.5190	0.0034
Rough Collie vs. Chinese Crested Dog	0.2970	0.1200	0.7340	0.0500
Smooth Collie vs. Chinese Crested Dog	0.1740	0.0661	0.4570	0.0056
Smooth Collie vs. German Shepherd Dog	0.1450	0.0647	0.3260	0.0020
Smooth Collie vs. German Spitz Mittel	0.1860	0.0659	0.5280	0.0158
Smooth Collie vs. Labrador Retriever	0.3070	0.1345	0.7020	0.0363
Smooth Collie vs. mixed breed	0.2400	0.1038	0.5550	0.0102
Smooth Collie vs. other	0.3570	0.1709	0.7460	0.0406
Smooth Collie vs. Pembroke Welsh Corgi	0.1740	0.0595	0.5110	0.0143
Smooth Collie vs. Staff. Bull Terrier	0.1900	0.0731	0.4920	0.0080
Spanish Water Dog vs. German Shepherd Dog	0.3810	0.1991	0.7300	0.0282

Supplementary information: questionnaire items utilised in the study

1. Tail-chasing/spinning - The dog tries to catch its tail and spins. The dog may stop and stare at its tail or it may just spin fast or slow.
Does your dog chase his/her tail?

- I've never noticed this behaviour
- a few times during the dog's lifetime
- every once in a while (monthly-yearly)
- quite often (weekly-monthly)
- repeatedly (every other day-weekly)
- daily
- several times per day

2. Reflections, shadows and invisible things - The dog stares/chases reflections or shadows, or the dog
looks like it is trying to catch invisible things.
Does your dog pursue, snatch, or bite at reflections, shadows or invisible things?

- I've never noticed this behaviour
- a few times during the dog's lifetime
- every once in a while (monthly-yearly)
- quite often (weekly-monthly)
- repeatedly (every other day-weekly)
- daily
- several times per day

3. Licking - Dog may lick e.g. floors and walls
Does your dog lick different surfaces?

- I've never noticed this behaviour
- a few times during the dog's lifetime
- every once in a while (monthly-yearly)
- quite often (weekly-monthly)
- repeatedly (every other day-weekly)
- daily
- several times per day

4. Pattern/schematic (ex. circle, figure 8's) or "aimless" running/movement
Does your dog run/pace indoors/outdoors (e.g. in a kennel) in a fixated manner?

- I've never noticed this behaviour
- a few times during the dog's lifetime
- every once in a while (monthly-yearly)
- quite often (weekly-monthly)
- repeatedly (every other day-weekly)
- daily
- several times per day

5. Visual fixation behaviour - Dog may stare at one point for very long period, even if there is really nothing to see. A dog may stare at wall, ceiling etc.
Does your dog stare (into space) at a specific spot for long periods?

- ○ I've never noticed this behaviour
- ○ a few times during the dog's lifetime
- ○ every once in a while (monthly-yearly)
- ○ quite often (weekly-monthly)
- ○ repeatedly (every other day-weekly)
- ○ daily
- ○ several times per day

6. Drinking too much or repeatedly visiting the water bowl (you can answer even if you feel that your dog's drinking behaviour is normal)
Drinking too much or repeatedly visiting the water bowl: How much time spent?
How much time does your dog spend daily on behaviour related to the water bowl (drinking, standing near it etc)?

- ○ less than 5 min
- ○ 5-15 min
- ○ 15min - 30 min
- ○ 30 min - 1 hour
- ○ 1 hour or more

7. Does your dog bite themselves (paws, flank or other body part)?

- ○ Never
- ○ Sometimes
- ○ Almost every day
- ○ Several hours per day

In questionnaire items 1-5, a dog was categorised into the low group if the owner answered “I've never noticed this behaviour” or “a few times during the dog's lifetime”. A dog was categorised into the high group if they performed the behavior “repeatedly (every other day-weekly)” or more often.

In questionnaire item 6, a dog was categorised into the low group if the owner answered “less than 5 min” and into the high group if they spent “15min - 30 min” or more near the water bowl.

In questionnaire item 7, a dog was categorised into the low group if the owner answered “Never” and into the high group if the owner answered “Almost every day” or “Several hours per day”.

If a dog was categorised into the high group in at least one questionnaire item (subtrait), it was categorised into the high group in “repetitive behaviour” trait. In contrast, only if a dog was categorised into the low group in all subtraits, it was categorised into the low group in “repetitive behaviour” trait.
