# Supplementary material for: Aggressiveness, ADHD-like behaviour, and environment influence repetitive behaviour in dogs
Source: Sci Rep. 2022 Mar 24;12:3520. doi: 10.1038/s41598-022-07443-6 (PMC8948230; doi:10.1038/s41598-022-07443-6)
Supplement: Supplementary file 2 — Supplementary Information 2. [file 41598_2022_7443_MOESM2_ESM.pdf]

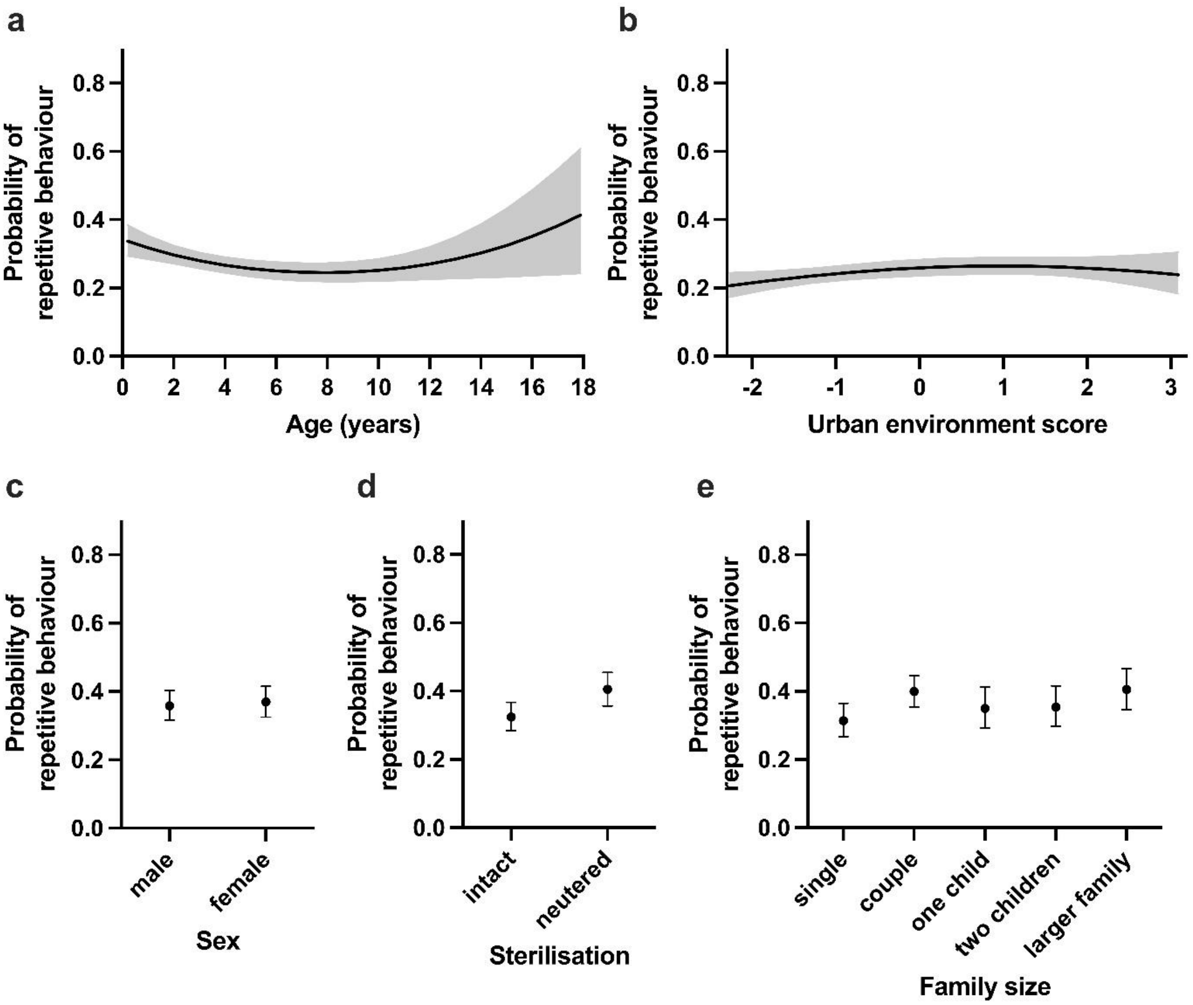

Supplementary Figure S1. The effects of age (**a**), urban environment score (**b**), sex (**c**), sterilisation (**d**), and family size (**e**) on the probability of repetitive behaviour in the logistic regression analysis. Grey area (**a, b**) and error bars (**c, d, e**) indicate 95% confidence limits. N = 4,436.
